# From Synthesis to Biological Impact of Pd (II) Complexes: Synthesis, Characterization, and Antimicrobial and Scavenging Activity

**DOI:** 10.1155/2016/4359375

**Published:** 2016-03-16

**Authors:** Nitin Kumar Sharma, Rakesh Kumar Ameta, Man Singh

**Affiliations:** School of Chemical Sciences, Central University of Gujarat, Gandhinagar 382030, India

## Abstract

The Pd (II) complexes with a series of halosubstituted benzylamine ligands (BLs) have been synthesized and characterized with different spectroscopic technique such as FTIR, UV/Vis, LCMS, ^1^H, and ^13^C NMR. Their molecular sustainability in different solvents such as DMSO, DMSO : H_2_O, and DMSO : PBS at physiological condition (pH 7.2) was determined by UV/Vis spectrophotometer. The* in vitro* antibacterial and antifungal activities of the complexes were investigated against Gram-positive and Gram-negative microbes and two different fungi indicated their significant biological potential. Additionally, their antioxidant activity has been analyzed with DPPH^•^ free radical through spectrophotometric method and the result inferred them as an antioxidant. The stronger antibacterial and antioxidant activities of the synthesized complexes suggested them as a stronger antimicrobial agent. Our study advances the biological importance of palladium (II) amine complexes in the field of antimicrobial and antioxidant activities.

## 1. Introduction

After a tremendous discovery of cisplatin, the synthesis and biological evaluation of new transition metal-based compounds are fields of growing interest [1]. The palladium (II) as nonplatinum metal complexes highly attracted the researchers because of its significant biological activity as well as lower side effects along with higher lipophilicity or solubility compared to cisplatin [2–5]. Palladium metal is a suitable candidate for metallodrugs because it displays structural properties similar to those of platinum and also exhibits promising* in vitro* cytotoxicity. Numerous Pd (II) complexes with different benzylamine ligands have been synthesized and their interesting* in vitro* biological activities have been reported [6, 7]. The antimicrobial activity of different palladium (II) complexes on the growth and metabolism of various groups of microorganisms has been studied and reported elsewhere. Garoufis et al. reviewed numerous scientific papers on antiviral, antibacterial, and antifungal activity of Pd (II) complexes with different types of ligands (sulfur and nitrogen donor ligands, Schiff base ligands, and different drugs as ligands) [8]. There are other interesting works which are reported recently in the literature showing different intensities of palladium complex activity on various species of bacteria and fungi [9–15]. In view of the growing cases of drug resistance of microorganisms it is urgent to search for more biospecific antifungal, less toxic agents. Metal-based drugs might answer this claim, representing an alternative therapeutic route. In this context, the discovery of nonplatinum based metal complexes came into consideration [16].

The aim of this paper is to synthesize new series of palladium (II) complexes and evaluate their* in vitro* antibacterial and antifungal activities against different microbes. The main aim of this research is focused on the biological impact of the newly synthesized Pd (II) complexes on different microorganism like Gram-positive and Gram-negative bacteria and different fungi. Hence, our study is an attempt to get overcome from microbial disease up to some extent. So with the aforesaid objectives we have synthesized new Pd (II) with halo substituted BLs and analyzed* in vitro* antimicrobial, antifungal, and antioxidant activity.

## 2. Experimental Section

### 2.1. Materials and Methods

Palladium dichloride (PdCl_2_), benzylamine ligands (BLs), DMSO, and ethanol (>99.5%) were purchased from Sigma Aldrich and used without further modifications. Elemental analysis was made with a Euro vector CHN analyzer, and UV/Vis spectra were recorded with a Spectro 2060 plus spectrophotometer over 200–600 nm in a 1 cm path length cuvette. FTIR (Perkin Elmer) spectra were taken with KBr palate where polystyrene thin film was used as a calibration standard. ^1^H and ^13^C NMR spectra were recorded in DMSO-d6 (NMR, 99.99%) with a Bruker-Biospin Avance-III 500 MHz FT-NMR spectrometer. Mass spectra were obtained with PE SCIEX API 165 with +*ve* ESI mode with ammonium acetate and acetonitrile in 1 : 9 v/v ratio as mobile phase. The molecular sustainability of Pd (II) complexes was determined by preparing a solution in DMSO, DMSO : water, and DMSO : phosphate buffer of pH 7.2. Buffer solution was prepared by adding 70 mL 0.1 M aqueous NaOH to 0.1 M aqueous KH_2_PO_4_ solution. The pH of a resultant buffer was checked with RS-232 modelled CyberScan pH 2100, EUTECH pH meter.

### 2.2. General Consideration for Synthesis

Initially, PdCl_2_ and BLs (molar ratio 1 : 2, resp.) were separately dissolved in freshly prepared solvent (absolute ethanol and Milli-Q water in 1 : 1.5) using 1 MLH magnetic stirrer. Then, the BLs solutions were added dropwise to metal compound solution with continuous stirring at room temperature. After 10 h, the mixture turned from light red brown to greenish color and after 16 h, precipitates were formed. The precipitates were filtered off, washed several times with chilled water/ethanol in 1 : 1 ratio, and kept overnight in vacuum oven at room temperature for absolute dryness.

### 2.3. Characterization Data

#### 2.3.1. Complex 1: C_14_H_15_N_2_Cl_3_Pd [Pd2CBA]

*Yield*: 0.1492 g, 67.615%.* Elemental analysis*, found: C, 39.65; H, 3.57; N, 6, 61%. Calcd for C_14_H_16_N_2_Cl_2_Pd: C, 40.01; H, 3.98; N, 7.88%.* IR *(*KBr*): *υ*_max/cm^−1^_ 3273 and 3226 (NH_2_), 1497 and 1457 (Ph, C=C), 756.46 (mono substituted Ph), 1075 (C–N), 567.4 (Pd–N), 405.9 (Pd–Cl). ^*1*^ *H NMR* (500 MHz; DMSO-d_6_; Me_4_Si) *δ* 3.74–3.80 (2H, s, Ph*CH*_*2*_NH_2_), 4.06–4.10 (2H, s, PhCH_2_N*H*_*2*_), 7.43–7.45 (1H, d, Ph*H*,* J* = 7.3 Hz), 7.72–7.75 (1H, d, Ph*H*,* J* = 6.5 Hz) and 7.32–7.36 (1H, t, Ph*H*). ^*13*^ *C NMR* (125 MHz; DMSO-d_6_; Me_4_Si) *δ* 45.43 (C1), 129.0 (C5), 129.2 (C4), 130.4 (C7), 132.2 (C3), 135.5 (C2), and 172.6 (C6). +*ve ESI-MS*: 424.93 *m*/*z* [M + 1] (calc. for [C_14_H_13_N_2_Cl_2_Pd] = 424.03).* UV/Vis* in DMSO: *λ*_max_ [ɛ (dm^3^mol^−1^cm^−1^)] = 275 (2066), 335 (265), 385 (221) nm, in DMSO : water (1 : 1): *λ*_max_ [ɛ (dm^3^mol^−1^cm^−1^)] = 265 (2465), in DMSO : phosphate buffer (1 : 1): *λ*_max_ [ɛ (dm^3^mol^−1^cm^−1^)] = 260 (1514), 335 (288) nm.

#### 2.3.2. Complex 2: C_14_H_15_N_2_Cl_3_Pd [Pd3CBA]

*Yield*: 0.1492 g, 67.615%.* Elemental analysis*, found: C, 39.24; H, 3.66; N, 6.43%. Calcd for C_14_H_15_N_2_Cl_3_Pd: C, 39.9; H, 4.11; N, 6.88%.* IR *(*KBr*): *υ*_max/cm^−1^_ 3273 and 3226 (NH_2_), 1497 and 1457 (Ph, C=C), 756.46 (mono substituted Ph), 1075 (C–N), 567.4 (Pd–N), 405.9 (Pd–Cl). ^*1*^ *H NMR* (500 MHz; DMSO-d_6_; Me_4_Si) *δ* 3.61–3.64 (2H, s, Ph*CH*_*2*_NH_2_), 4.06–4.10 (2H, s, PhCH_2_N*H*_*2*_), 7.34–7.36 (1H, d, Ph*H*,* J* = 7.3 Hz), 7.43–7.45 (1H, d, Ph*H*,* J* = 6.5 Hz) and 7.57–7.59 (1H, s, Ph*H*). ^*13*^ *C NMR* (125 MHz; DMSO-d_6_; Me_4_Si) *δ* 45.59 (C1), 129.74 (C5), 128.5 (C4), 129.4 (C7), 133.2 (C3), 136.5 (C2), and 174.6 (C6). +*ve ESI-MS*: 424.93 *m*/*z* [M + 1] (calc. for [C_14_H_13_N_2_Cl_2_Pd] = 424.03).* UV/Vis* in DMSO: *λ*_max_ [ɛ (dm^3^mol^−1^cm^−1^)] = 275 (2066), 335 (265), 385 (221) nm, in DMSO : water (1 : 1): *λ*_max_ [ɛ (dm^3^mol^−1^cm^−1^)] = 265 (2465), in DMSO : phosphate buffer (1 : 1): *λ*_max_ [ɛ (dm^3^mol^−1^cm^−1^)] = 260 (1514) nm.

#### 2.3.3. Complex 3: C_14_H_15_N_2_Cl_3_Pd [Pd4CBA]

*Yield*: 0.1492 g, 67.615%.* Elemental analysis*, found: C, 39.88; H, 3.84; N, 6.66%. Calcd for C_14_H_15_N_2_Cl_3_Pd: C, 40.22; H, 4.93; N, 6.88%.* IR *(*KBr*): *υ*_max/cm^−1^_ 3273 and 3226 (NH_2_), 1497 and 1457 (Ph, C=C), 756.46 (mono substituted Ph), 1075 (C–N), 567.4 (Pd–N), 405.9 (Pd–Cl). ^*1*^ *H NMR* (500 MHz; DMSO-d_6_; Me_4_Si) *δ* 3.58–3.61 (2H, s, Ph*CH*_*2*_NH_2_), 4.00–4.03 (2H, s, PhCH_2_N*H*_*2*_), 7.37–7.39 (1H, s, Ph*H*) 7.50 (1H, d, Ph*H J* = 8.43 Hz,* 1H*) 7.49–7.51 (1H, d, Ph*H*,* J* = 8.23 Hz* 1H*). ^*13*^ *C NMR* (125 MHz; DMSO-d_6_; Me_4_Si) *δ* 45.49 (C1), 129.69 (C5), 129.47 (C4), 131.6 (C7), 131.4 (C3), 136.5 (C2), and 173.6 (C6). +*ve ESI-MS*: 424.93 *m*/*z* [M + 1] (calc. for [C_14_H_13_N_2_Cl_2_Pd] = 424.03).* UV/Vis* in DMSO: *λ*_max_ [ɛ (dm^3^mol^−1^cm^−1^)] = 275 (2066), 335 (265), 385 (221) nm, in DMSO : water (1 : 1): *λ*_max_ [ɛ (dm^3^mol^−1^cm^−1^)] = 265 (2465) nm, in DMSO : phosphate buffer (1 : 1): *λ*_max_ [ɛ (dm^3^mol^−1^cm^−1^)] = 265 (1514) nm.

### 2.4. UV/Vis Spectroscopy

Electronic spectra were recorded with a Spectro 2060 plus model UV/Vis spectrophotometer from 200 to 600 nm using 1 cm path length cuvette. DMSO was used for solution preparation. The stability of compounds was determined by preparing a solution in DMSO, DMSO/water, and DMSO/phosphate buffer of pH 7.2. Buffer solution was prepared by adding 70 mL 0.1 M aqueous NaOH solution to 0.1 M aqueous KH_2_PO_4_ solution. The pH of the resultant buffer was checked with RS-232 modelled CyberScan pH 2100, EUTECH pH meter instrument. Their concentration for the UV study with DMSO, DMSO : water, and DMSO : PBS was kept constant at 1 × 10^−3 ^M.

### 2.5. HRTEM Images and SAED Pattern

The TEM images of the Pd2MBA were taken by HRTEM Jeol Jem 2100 at different magnification. The sample analysis was done by dispersing the sample in water and then dropped on carbon coated copper grid. After complete dryness of the sample on grid, the grid was inserted in specimen and then the images were taken.

## 3. Biological Evaluation

### 3.1. Microorganism Test

The synthesized palladium (II) complexes tested against 6 microbes for their biological potential. They screened their antibacterial activities against human pathogenic bacteria, namely, Gram-negative (*Escherichia coli*; NCIM 2109 and* Pseudomonas aeruginosa*; NCIM 2036) and Gram-positive (*Staphylococcus aureus*; NCIM 2079 and* Bacillus subtilis*; NCIM 2250) bacterial strains and two fungal strains (*Candida albicans*; NCIM 3471 and* Aspergillus niger*; NCIM 545) by Kirby Beurs Disc Diffusion Method using DMSO as solvent at 200 *μ*gmL^−1^ on Mueller Hinton Agar media. The zone of inhibition was measured in millimetre (mm) after 24 h incubation at 37°C and pH 7.4. The zones of inhibition were compared with the standard drugs* chloramphenicol* (10 *μ*g) and* ciprofloxacin* (10 *μ*g). Discs with only DMSO were used as positive control.

### 3.2. Antioxidant Activities

Antioxidant activities have been studied on free radical scavenging of stable 1-2, 5- diphenyl-2-picrylhydrazyl (DPPH^•^). For this purpose stock solution of complexes and DPPH^•^ (0.002%) were mixed in DMSO + water (1 : 1) for Pd (II) complexes. For sample preparation, the DPPH^•^ solution was mixed with a complex solution in 1 : 1, followed by vigorous shaking, and thereafter kept for incubation of 30 min in dark. The UV absorbance was measured at 517 nm with UV/Vis spectrophotometer and a decrease in DPPH^•^ absorbance was noted which indicates a radical-scavenging activity calculated with the following equation:

(1) $$\begin{array}{c} \text{Scavenging activity}\%=\left(\;\frac{A_{0}-A_{s}}{A_{0}}\;\right)\times 100. \end{array}$$

*A*_*S*_ is absorbance of DPPH^•^ with a test compound and *A*_0_ is absorbance of DPPH^•^ without a test compound. Absorbance data are presented as means ± SD of three determinations.

## 4. Results and Discussion

### 4.1. Synthesis and Characterization

**Figure 1:**
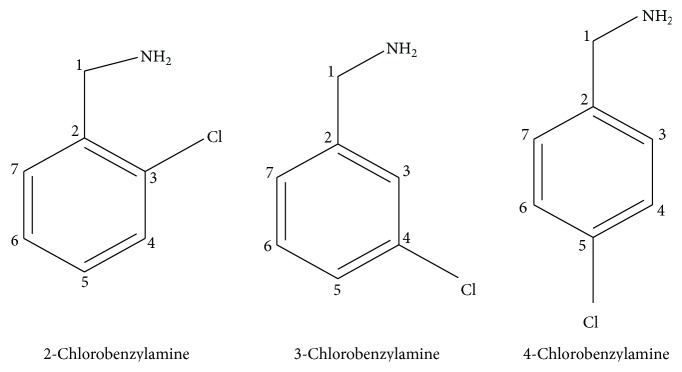
Structures of the ligands (L = BLs).

PdCl(BLs)_2_ have been synthesized allowing reaction of PdCl_2_ with different BLs (Figure 1) in 1 : 2 molar ratio over 16 h as per Reaction Scheme. The ethanol + water solution in 1 : 1.5 ratios was used for the synthesis of all the complexes solution.

*Reaction Scheme*. Synthesis of Pd (II) complexes is as follows:

(2) $$\begin{array}{c} {PdCl}_{2}+2BLs\overset{\text{Aqueous }C_{2}H_{5}OH}{\underset{16\;h/rt}{\to }}PdCl{\left(\;BLs\;\right)}_{2} \end{array}$$

**Figure 2:**
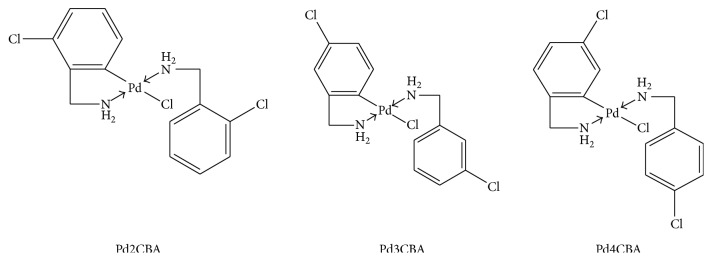
Structure of synthesized Pd (II) complexes.

The 3300 to 3119 cm^−1^ stretching frequencies inferred presence of NH_2_ of benzylamine ligands in the complexes and similarly from 1497 to 1453 cm^−1^ predicted C=C in phenyl ring. The 495.92 to 438.78 cm^−1^ and 380–348 cm^−1^ bands indicate the Pd–N and Pd–Cl bands, respectively [17, 18]. In ^1^H NMR, 2H of –N*H*_*2*_ and PhC*H*_*2*_– appeared at *δ* 4.03 to 4.10 and 3.58 to 3.80, respectively, with singlet for all complexes. The aromatic protons appeared with their specific peak from *δ* 7.32 to 7.59 (*J* = 7 to 8). In ^13^C NMR, the benzyl carbon (Ph*C*H_2_–) at *δ* 45.43 to 43.59 ranges for all the (PdCl(BLs)_2_) complexes [19]. The aromatic ortho, meta, and para –Cl attached carbon appeared within *δ* 145.722 to 128.6 for the complexes. The carbon of –Cl at ortho, meta, and para appeared at *δ* 132.2, 128.5, and 129.69 ppm, respectively. The +ve ESI mass spectra of Pd complexes have found [M + 1] confirming their molecular mass. The UV/Vis absorption from 265 to 270 nm and ^1^H NMR coupling constant between 5 and 9 MHz have confirmed their* trans* geometry (Figure 2) [20–22].

### 4.2. Absorption Spectroscopy

**Figure 3:**
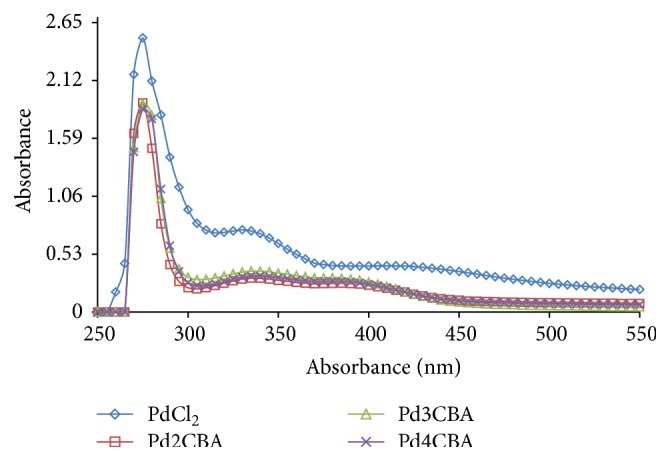
UV spectra of Pd (II) complexes in DMSO at 0.001 M.

**Figure 4 fig4:**
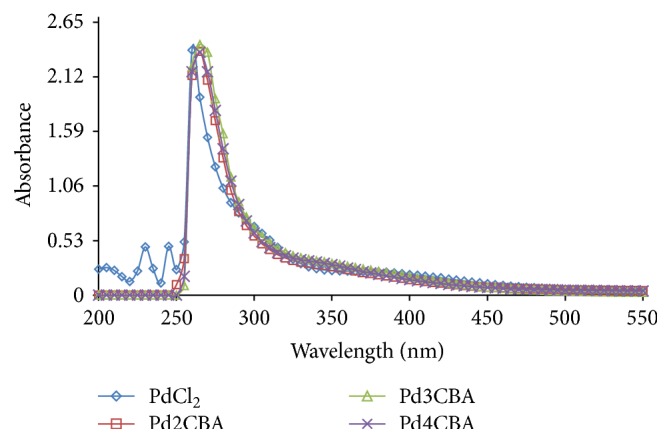
UV spectra of Pd (II) complexes in DMSO : water.

**Figure 5:**
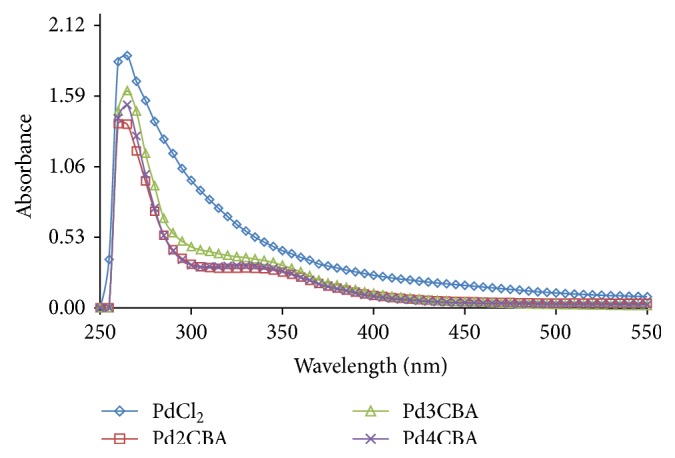
UV spectra of Pd (II) complexes in DMSO : phosphate buffer at 7.2 pH.

To investigate a solid state structure retained in solution, the UV/Vis spectral behaviour was investigated in DMSO and DMSO + water as well as in DMSO + phosphate buffer for PdCl(BLs)_2_. The overall patterns of spectra for complexes solution were found similar to different mediums to ensure their molecular sustainability in different solvents. The absorption spectrum consists of a band at 400 nm and may be assigned as 1A_1 g_ → 1A_2 g_ (*d*_*xy*_ → *d*_*x*^2^−*y*^2^_) transition occurring with Pd complexes (Figures 3 [Fig fig4]–5) [23]. The UV/Vis absorption band from 265 to 270 nm has confirmed their* trans* geometry of the PdCl(BLs)_2_ [20–22].

### 4.3. SAED Pattern through HRTEM

**Figure 6:**
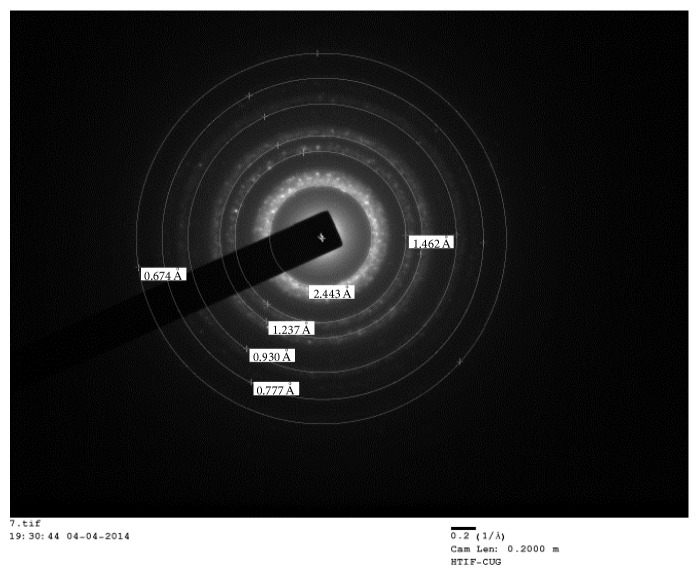
SAED pattern of Pd2MBA.

The TEM images reports from the SAED pattern were taken at different magnification suggesting the presence of different elements which are semicrystalline and homogeneous in shape (Figure 6, ESI Figures S1 and S2 in Supplementary Material available online at http://dx.doi.org/10.1155/2016/4359375). The proper alignment is not seen in the rings which indicate that the complex is not completely crystalline but a clear formation of rings suggesting the semicrystalline nature of complexes.

### 4.4. Biological Potential

#### 4.4.1. Antimicrobial Activity

**Figure 7:**
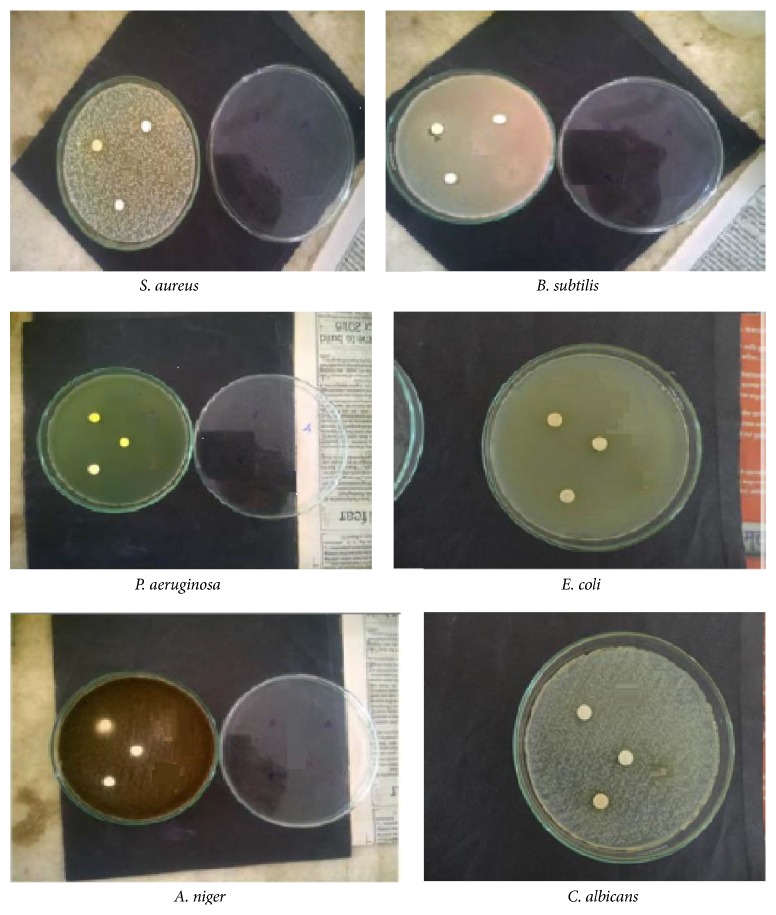
Antimicrobial studies of Pd (II) complexes.

Biological evaluation was made by Kirby Beurs Disc Diffusion Method as per standard procedure [24, 25]. The PdCl(BLs)_2_ gave best response against Gram-negative (*E. coli* and* P. aeruginosa*) and Gram-positive (*S. aureus* and* B. subtilis*) microbes (Figure 7, Table 1). All Pd (II) complexes are not effective against the fungal microbes (*C. albicans* and* A. niger*) and they have not shown any zone of inhibition. The biological evaluation inferred that the PdCl(BLs)_2_ showed greater activity against positive organisms and less activity against negative organisms.

#### 4.4.2. Antioxidant Activities

**Figure 8:**
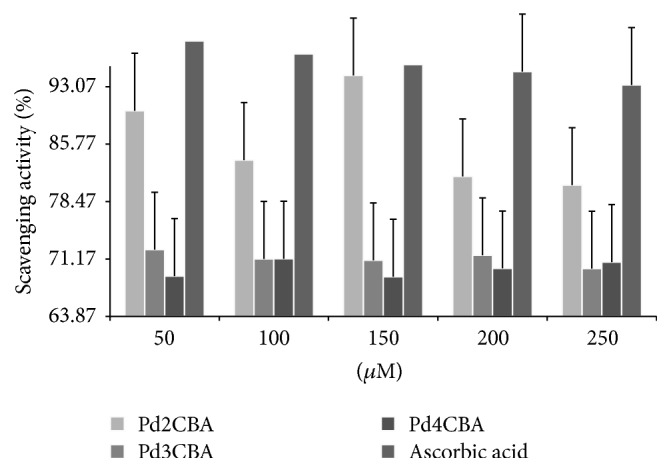
Free radical-scavenging activities of synthesized complexes.

The scavenging activities have been investigated to support the biological potential of PdCl(BLs)_2_ [26, 27]. The antioxidant activities have been studied with analyzing the decrease in absorbance or scavenging effect of a stable free DPPH^•^ as per standard procedure for the PdCl(BLs)_2_ [28, 29]. The percentage scavenging activity of Pd (II) complexes has been determined in a concentration-dependent mode in comparison to the DPPH^•^ absorption at 517 nm [30–32]. The DPPH^•^ free radical's absorption at 517 nm with DMSO was 0.906 for Pd (II) complexes. From 50 to 250 *μ*M with an interval of 50 *μ*M, complexes have expressed a decrease in absorption (Figure 8) that characterized them as an antioxidant.

The highest 94.49, 72.36, and 70.79% for Pd2CBA, Pd3CBA, and Pd4CBA, respectively, inferred Pd2CBA a strong antioxidant among them. The obtained values also compared with the control ascorbic acid which inferred that the results are very closer to control range. Thus, the antioxidant activities of Pd complexes have inferred their significance in medicinal as well as material sciences.

## 5. Conclusion

Genome studies have provided a better understanding of the closer distance between the microbial kingdom and the human species. The synthesized PdCl(BLs)_2_ complexes showed selective and moderate activity against different microbes and the interesting results were obtained for Gram-positive species, which are common in the environment. Apart from their microbial studies, the complexes have also expressed significant free radical-scavenging activities acting as antioxidants and could be used for medicinal purposes. However, in present study, we have focused on their antimicrobial and antioxidant activities and their other activities relevant to medical, biophysical, and biochemical processes are being pursued. The anticancer activity on suitable cell line against solid tumours, apoptosis, and DNA binding studies are under progress.

## Supplementary Material

The supplementary Information (ESI) contains ESI Figure S-1 and S2 which represent the HRTEM images of Pd2CBA at different magnifications.

## Figures and Tables

**Table 1:** Antimicrobial activity of Pd (II) complexes.

Complexes	Gram-positive bacteria		Gram-negative bacteria		Fungi (yeast)	Fungi (mould)
	*Staphylococcus aureus*	*Bacillus subtilis*	*Escherichia coli*	*Pseudomonas aeruginosa*	*Candida albicans*	*Aspergillus niger*
Pd2CBA	5.25	7.76	7.98	6.99	—	—
Pd3CBA	6.98	8.23	9.1	8.09	—	—
Pd4CBA	7.79	8.15	8.76	6.27	—	—
Chloramphenicol	28.67	24.44	29.63	26.30	NA	NA
Ciprofloxacin	21.11	22.23	22.33	21.34	NA	NA
Amphotericin-B	NA	NA	NA	NA	14.23	15.34
