# Supplementary material for: From Synthesis to Biological Impact of Pd (II) Complexes: Synthesis, Characterization, and Antimicrobial and Scavenging Activity
Source: Biochem Res Int. 2016 Mar 16;2016:4359375. doi: 10.1155/2016/4359375 (PMC4812500; doi:10.1155/2016/4359375)
Supplement: Supplementary file 1 — The supplementary Information (ESI) contains ESI Figure S-1 and S2 which represent the HRTEM images of Pd2CBA at different magnifications. [file 4359375.f1.zip › mat.4359375.v2/ESI Figure 2.docx]

**Figure S1.** HRTEM images of Pd2MBA at 250000x magnification
